# Psychotropics, Antidepressants, and Visceral Analgesics in Functional Gastrointestinal Disorders

**DOI:** 10.1007/s11894-018-0664-3

**Published:** 2018-11-05

**Authors:** Hans Törnblom, Douglas A. Drossman

**Affiliations:** 1 Department of Internal Medicine & Clinical Nutrition, Institute of Medicine, Sahlgrenska Academy, University of Gothenburg, SE-41345 Gothenburg, Sweden; 2 Center for Functional Gastrointestinal and Motility Disorders, University of North Carolina, Chapel Hill, NC USA; 3 Center for Education and Practice of Biopsychosocial Care and Drossman Gastroenterology, Chapel Hill, NC USA

**Keywords:** Functional gastrointestinal disorders, Disorders of brain-gut interaction, Multidimensional clinical profile, Treatment, Antidepressants, Abdominal pain

## Abstract

**Purpose of Review:**

The functional gastrointestinal disorders, or disorders of gut-brain interaction as defined by the Rome IV criteria, are the most common diagnostic entities in gastroenterology. Treatments that address the dysregulation of gut-brain interaction with these disorders are increasingly gaining interest as a better option than for example traditional analgesics, particularly opioids. Antidepressants, antianxiety and antipsychotic medications, and visceral analgesics, now termed neuromodulators, are included in this update addressing the evidence of treatment benefit in disorders of brain-gut interaction.

**Recent Findings:**

By a careful selection based on a multidimensional clinical profile, a decreased symptom burden, particularly regarding abdominal pain, nausea, and vomiting, as well as improved social function and quality of life, can be obtained by use of neuromodulators. There is good evidence for the peripheral neuromodulators from studies in bowel disorders, and the central neuromodulators both from indirect evidence in chronic pain disorders as well as selected disorders of brain-gut interaction.

**Summary:**

Basic knowledge about the pharmacologic properties and clinical use of neuromodulators in disorders of brain-gut interaction improves the treatment outcome and avoids use of traditional analgesics.

## Introduction

The functional gastrointestinal disorders (FGIDs) are currently defined by the Rome IV criteria [1] and include 33 different diagnostic entities in adults where neurogastroenterological interactions are increasingly highlighted as a central pathophysiologic mechanism. In fact, the terminology has changed due to this into naming FGIDs as disorders of gut-brain interactions (DGBIs). There is now good evidence that treatment modalities addressing this association should be an integral part of the approach to the patient who experiences troublesome DGBI symptoms. In particular, this includes abdominal pain and closely related symptoms such as nausea and vomiting. In order to avoid the stigma connected with some of the pharmacologic treatment modalities, such as antidepressants, antipsychotics, or other psychotropic terminologies, a recent Rome Foundation working team report [2••] introduced the term neuromodulators with the intention to put more focus on the neurologic interaction relating to treatment, rather than the historical term targeted at psychiatric disorders. There is also good reason to distinguish from a pharmacologic point of view those treatments that have predominant peripheral effects, i.e., effects on the enteric nerve system, those treatments with predominant central effects on the central nervous system, and those treatments where there are combined effects. With the use of the term neuromodulators, we extend the actions of these compounds that are outside of the range defined by medications used within psychiatry.

The scope of this article is to update recent knowledge gained about the use of neuromodulators in DGBIs and to put it into perspective for clinical practice, to optimize the patient-doctor interaction and to avoid misuse of some treatment options for abdominal pain, mainly opioids that might cause drug dependence and narcotic bowel syndrome [3].

## Multidimensional Clinical Profile

**Fig. 1:**
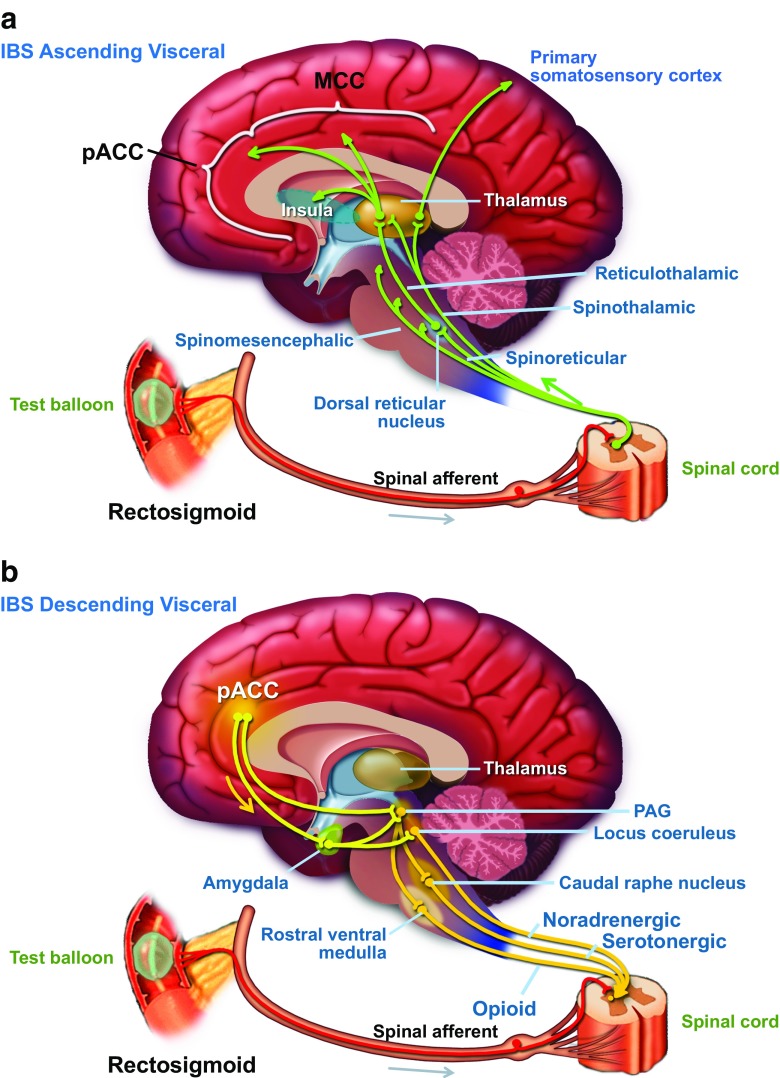
Simplified overview of ascending (**a**) and descending (**b**) neural pathways involved in gut-brain interactions, mainly perception and pain regulation. The descending modulatory fibers from brain stem centers can alter the sensitivity of the dorsal horn neuron signaling and can serve as a central control of pain perception during visceral stimulation. Used with permission from the Rome Foundation

A good starting point when treatment involves one or more neuromodulator is to thoroughly characterize the patient’s illness by understanding the full extent of the illness experience and in doing so predict which dimension of ill health might improve from specific treatment options. This concept is supported by expert consensus where an in-depth description including clinical examples can be found in an updated version as part of the Rome IV process [4•]. Briefly, the concept includes five dimensions and the complexity of the illness and its specific treatment is determined by the interaction of these influencing dimensions: Rome IV-based diagnosis, additional sub-classifications that might differentially affect treatment such as bowel habits in IBS, patient-defined impact of the illness on daily life, psychosocial modifiers of relevance, and finally physiologic modifiers of function such as a transit test or biomarkers. This type of assessment ultimately can improve patient outcome in situations where multiple factors contribute to the illness. A key factor is also to help the patient understand why neuromodulators that have effects on different aspects of the gut-brain axis can be useful in their care. This concept is important as it helps explain factors involved in a biopsychosocial interaction with multiple contributors to the illness. A simplified neuroanatomical picture, such as Fig. 1a, b, helps patients understand where and how a particular neuromodulator will have effects. An in-depth implementation guide for this is included in the recent Rome Foundation working team report outlining recommendation for neuromodulators in FGIDs [2••].

## Peripheral Neuromodulators

The treatment options that follow in this section may not traditionally be considered neuromodulators. However, they are included for their heuristic value in keeping with the concept of having peripheral (i.e., visceral) action on gut neuromuscular or sensory function via the enteric nervous system.

### Antispasmodics

This class of drugs can be considered as having neuromodulator effects mainly by many of them having anticholinergic properties. Visceral smooth muscle spasm has been considered as a putative, but non-proven, mechanism for the pain component in some DGBIs, most often IBS, which has driven the clinical use. Older studies, not well-designed and heterogeneous, have yielded results in meta-analyses indicating that antispasmodics as a group are in general superior to placebo [5]. Peppermint oil is included in this group of neuromodulators, and the _L_-menthol ingredient is indicated as being a κ-opioid receptor agonist [6] and 5-hydroxytryptamine (HT)_3_ receptor antagonist [7] also suggesting visceral analgesic effects. Recently, a slow-release preparation of peppermint oil was evaluated in a randomized, controlled study showing superiority over placebo both measured by a total IBS symptom score as well as in individual symptom scores such as abdominal pain, urgency, bloating, and distension [8].

### Guanylate Cyclase-C Receptor Agonists

One recent type of visceral analgesic relates to the functional effects of guanylate cyclase-C (GC-C) receptor stimulation in the gut [9]. The analgesic properties are mediated by a cascade of intracellular events starting with GC-C stimulation that gives rise to intracellular cyclic guanosine monophosphate (cGMP) production. cGMP transported to the extracellular space modulates the conduction properties of nociceptive neurons located in the submucosa. This concept is based on mechanistic studies in animal models and has been shown to be relevant in the treatment of the pain component of IBS [10]. The effect of GC-C stimulation on fluid excretion results in an accelerated gut transit that restricts its use to those patients with a bowel habit dominated by constipation. Linaclotide was the first substance available for clinical use after proving to be effective in IBS-C [11•, 12], followed by plecanatide [13] that is available at some markets with the same indication. The effects on abdominal pain have been shown to develop gradually during the first 2 months of treatment, after which a bit less than half of the studied populations meet the FDA end-point for pain relief (improvement of ≥ 30% in the worst abdominal pain score vs average baseline pain) [11•].

### Peripheral Opioid Receptor Agonists/Antagonists

Stimulation of the visceral μ-receptor has for long been the first-line treatment option in conditions involving chronic diarrhea, including IBS-D. From the historical use of opioids with both peripheral and central effects, the advent of loperamide, a μ-receptor agonist that does not penetrate the blood-brain barrier was a major break-through. One limiting factor has been that a substantial proportion of patients do not tolerate this treatment due to the development or aggravation of abdominal pain or constipation. A new therapeutic option, eluxadoline, has agonistic properties on μ- and κ-receptors, and antagonistic properties on δ-receptors. Animal studies showed promise that eluxadoline could reduce visceral hypersensitivity [14], one of the key pathophysiologic mechanisms in IBS, and normalize transit in diarrhea models where the combined μ- and δ- receptor effects normalized transit over a wider dose range compared with loperamide [15]. Due to a limited bioavailability after oral administration [16], the central effects of opioid receptor stimulation are avoided and clinical trials with treatment given for up to 52 weeks have not shown signs of abuse potential or opioid withdrawal effects after treatment termination [17]. The clinical effects on the key symptoms of IBS-D in two large-scale phase III trials show superiority compared to placebo as assessed by reduction in abdominal pain and improved stool form after 12 and 26 weeks of treatment for both doses studied (75 and 100 mg BID) [18•]. The specific effects on abdominal pain still need further study since the reduction in the abdominal pain domain of the composite primary end-point is less convincing and not significantly better than placebo. Furthermore, the issue of an increased occurrence of pancreatitis associated with eluxadoline treatment, both in the pivotal study [18•] and in post-marketing surveillance [19], warrants careful consideration of known risk factors for pancreatitis in general among those patients with IBS-D considered for treatment. At this point of time, analysis of the FDA Adverse Event Reporting System (FAERS) also highlights that those who have had a cholecystectomy or are suffering from previous or current serious liver disease must be included in the group of patients where eluxadoline should not be used.

### Serotonin Receptor Agonists/Antagonists

Serotonin receptor modulation is of interest in treatment of DGBIs, mainly due to effects on GI motor function, where 5-HT_3_ receptor antagonists slow, and 5-HT_4_ receptor agonists accelerate oro-anal transit. These local neuromodulator effects on gut function are useful to treat symptoms of diarrhea and constipation respectively. Studies of alosetron, a 5-HT_3_ receptor antagonist, have also shown improvement in other key symptoms in female patients with IBS-D, such as abdominal pain, discomfort, and bloating [20]. Despite initial serious side effects from this class of drug (severe constipation, ischemic colitis), clinical experience after reintroduction with a restricted prescription program has yielded minimal complications, in part because clinicians are more aware of how to appropriately prescribe this medication. A recent prospective, open-label, follow-up study in multiple US centers prescribing alosetron to women with IBS-D showed no serious side effects with a 0.5 mg BID regimen [21]. In Asia, another 5-HT_3_ receptor antagonist, ramosetron, is approved for the treatment of IBS-D in both men and women and with similar effects both on bowel function and abdominal pain and discomfort [22]. Unlike alosetron and ramosetron, ondansetron given to IBS-D patients did not have a significant effect on abdominal pain in a study with crossover design [23].

Prucalopride, a selective 5-HT_4_ agonist, has as its main indication treatment of chronic constipation [24•, 25, 26] due to its effects on gastrointestinal transit resulting in increased numbers of bowel movements and constipation-associated symptoms in a wider sense. Pooled data from these phase III trials on the 2 mg per day dose in women shows that prucalopride has large or moderate positive effects on symptoms such as abdominal pain and discomfort, bloating, and painful bowel movements [27].

### Delta Ligand Agents

This class of drugs is classified as peripheral neuromodulators even if their blockage of the α_2_δ subunit of voltage-sensitive calcium channels on neurons exerts more general effects in nociceptive pathways. The evidence for use in DGBIs is low even if pregabalin has been shown to positively affect visceral sensory thresholds in experiments on IBS patients [28]. From a conceptual point of view, when the delta ligand agents are considered as treatments of DGBI symptoms, this can be looked upon as an indicator for considering use of a central neuromodulator with better evidence for effects. Special clinical situations, such as IBS with comorbid fibromyalgia [29] or pain with a clear component of abdominal wall origin, could justify the use of pregabalin (150–600 mg/day) on its own. Fibromyalgia-related and neuropathic pain mechanisms are being mediated by the same type of central sensitization as chronic pain in DGBIs.

## Central Neuromodulators

The central neuromodulators have effects on gut-brain interactions that are more widespread compared to the peripheral mechanisms discussed above. Visceral sensory input is conveyed by a chain of three orders of neurons that synapse at the dorsal root ganglion of the spinal cord and the thalamus, before reaching conscious perception in the primary somatosensory cortex (discrimination and localization), the reticular formation of the brainstem (emotional processing), the medial thalamus, the cingulate cortex, and the insula (behavioral response). Meta-analysis of brain-imaging studies shows the anterior cingulate cortex and insular regions as central for all painful modalities [30], but the network activation of larger brain regions, where the magnitude of response determines symptom experience has expanding data supporting it [31]. A central pain control mechanism also has the ability to modulate sensory perception via descending fibers from the brain stem that affects the transmitting properties at the level of the dorsal horn neuron (the first and second afferent neuron conduction site). There is also good evidence that neural plasticity involving neurodegenerative components is involved in particularly chronic pain [32–34] and that neuromodulators actually can have positive neuroplastic, regenerative effects [35•] when used for treatment. Taken together with basic knowledge about the main transmitters of information in the gut-brain axis, serotonin (5-HT), noradrenalin (NA), dopamine, and their receptor sites, the central modulators can be tailored for use in DGBIs. A summary of the different drug classes, their suggested clinical indications, and dosage can be found in Table 1.

**Table 1:** Summary of the central neuromodulators, their modes of action, suggested clinical indications, main side effects, and dosage in the treatment of disorders of gut-brain interactions (DGBIs)

Drug class	Mode of action	Clinical indications/evidence for effectiveness	Side effects	Drugs/dose
Tricyclic antidepressants	Pre-synaptic SRI and NRI. Antagonism/inhibition of multiple post-synaptic (5-HT_2_, 5-HT_3_, H_1_, M1, α_1_) and pre-synaptic (α_2_) receptors	Chronic abdominal pain in all DGBIs where abdominal pain is a prominent feature. Best documented for IBS, but also FD (EPS)	Drowsiness, dry mouth, constipation, sexual dysfunction, arrhythmias, weight gain	Amitriptyline, imipramine, desipramine, nortriptyline. 25–100 (– 150) mg qd for all
Selective serotonin reuptake inhibitors	Pre-synaptic SRI	Treatment of anxiety, phobic features, and OCD in all DBGIs. Not helpful for pain but evidence of improvement of global measures in IBS and upper chest pain	Agitation, diarrhea, insomnia, night sweats, headache, weight loss, sexual dysfunction	Citalopram (10–40 mg qd), escitalopram (5–20 mg qd), fluoxetine (10–40 mg qd), paroxetine (10–40 mg qd), sertraline (50–150 mg qd)
Serotonin and noradrenalin reuptake inhibitors	Pre-synaptic SRI and NRI	Useful for abdominal pain in DGBIs based on data for fibromyalgia, back pain, headache, and other chronic pain. Formal studies for DGBIs needed	Nausea, agitation, dizziness, sleep disturbance, fatigue, liver dysfunction (rare)	Duloxetine (30–90 mg qd), milnacipran (50–100 mg bid), venlafaxine (for pain 150–225 mg qd). NRI effective for all doses with duloxetine. NRI effective only in higher doses (> 150 mg) for venlafaxine. Milnacipram stronger NRI than SRI effects
Tetracyclic antidepressants	Indirect effects resulting in increased noradrenergic and serotonergic activity through α_2_ antagonism on noradrenergic and 5-HT neurons. Also 5-HT_2_, 5-HT_3_, H_1_, M1 antagonism	Treatment of early satiation, weight loss, and chronic nausea/vomiting. Side effect profile can be useful to improve sleep. Documentation mainly for FD (PDS)	Sedation, headache, dry mouth, weight gain	Mirtazapine (15–45 mg qhs), mianserin (30–90 mg qhs), trazodone (75–150 mg qhs)
Azapirones	Partial pre- and post-synaptic 5-HT_1_ agonists	Treatment of associated anxiety and FD (PDS). Potential use for treatment in other DGBIs	Sedation, headache, dizziness, vertigo	Buspirone (15–45 mg bid), tandospirone (10 mg tid)
Atypical antipsychotics	D2 receptor antagonism as main mechanism. Various profiles of 5-HT_2A_ antagonism (olanzapine, quetiapine), 5-HT_1A_ agonism (quetiapine), H_1_, α_1_, α_2_, M1 receptor antagonism	Potential use in augmentation for abdominal pain reduction and improved sleep (quetiapine and olanzapine). Further studies needed for DGBIs	Sedation, dizziness, and weight gain. Hyperlipidemia and diabetes	Apriprazole (2.5–7.5 mg qd), olanzapine (2.5–10 mg qd), quetiapine (25–200 mg qd)

*SRI* serotonin reuptake inhibition, *NRI* noradrenaline reuptake inhibition, *5-HT* 5-hydroxytryptamine, *H* histamine receptor, *M* muscarinic acetylcholine receptor, *FD* functional dyspepsia, *EPS* epigastric pain syndrome, *PDS* postprandial distress syndrome, *OCD* obsessive compulsive disorder, *IBS* irritable bowel syndrome

### Tricyclic Antidepressants

Tricyclic antidepressants (TCA) are the first-line pharmacologic treatment for symptom improvement in DGBIs where pain is a prominent feature. Their mode of action is by 5-HT and NA reuptake inhibition in combination with additional receptor antagonistic properties (5-HT_2A_ and _2C_, muscarinic_1_, histamine_1_). There are slight variations comparing different TCAs from these aspects where the tertiary amines (amitriptyline, imipramine) are more prone to produce side effects from their greater antimuscarinic and antihistaminic actions compared to the secondary amines (desipramine, nortriptyline). These side effects, particularly sedation and constipation, can be used also to treat some aspects of DGBIs such as sleep disturbance and diarrhea, if present.

Most studies of TCAs in DBGIs are performed in IBS populations where meta-analysis favors their positive effects in pain reduction with favorable numbers needed to treat [36]. Of notice is that there are no data in support of using a dose regimen below 25 mg/day, rather the dose range 25–100 mg/day (up to150 mg/day) is recommended where the treatment effect, or if anticholinergic side effects become bothersome, decides the final dose [2••]. TCAs are also useful to treat the pain component in those diagnosed with functional dyspepsia based on findings from a recently published study [37••], where the most obvious positive effects were to be found in those with ulcer-like dyspepsia (Rome II definition, considered as epigastric pain syndrome (EPS) with Rome IV). In the same study, it was also shown that escitalopram, an SSRI, did not have the same positive effect on abdominal pain as TCAs, thus supporting the more limited use of SSRIs for treating painful DGBIs due to their lack of NA effects.

Due to the effects TCAs have on cardiac fast sodium channels, there is a proarryhthmic potential that motivates a baseline ECG to check for risk factors (prolonged QT-interval, left bundle branch block, bifascicular block) and avoid their use in those that have had a myocardial infarction. Thus, it is important to consider that potential benefit with higher dosages of TCAs (particularly the tertiary amine agents) is compromised by their greater potential for side effects. In general, helping patients to overcome transient side effects when starting treatment with a central neuromodulator is important in order to avoid unnecessary treatment failures. Identifying nocebo effects from negative expectations [38•] and titrating the medication to effective doses are central actions to take care of and often warrant more frequent initial consultations and support before a positive circle of symptom reduction and trust in the treatment is established.

### Serotonin Noradrenalin Reuptake Inhibitors

Serotonin noradrenalin reuptake inhibitors (SNRIs) have somatic analgesic properties based on studies of neuropathic pain in diabetes, fibromyalgia, back pain, and headache [2••, 39], the latter three being common comorbid features in DGBIs. While there is no formal evidence for use in DGBIs, these findings can be extrapolated to those patients where abdominal pain is a prominent feature and has been recommended by a recent Rome Foundation working team review [2••]. SNRIs have an advantage in relatively few side effects comparing with TCAs and can be considered as an alternative when side effects from TCAs restrict their use, and also when constipation or comorbid depression is part of the illness. The most common side effect is nausea, which tends to diminish after the first week and is reduced when taken with food. In general, the NA reuptake inhibition properties that are the most important for the analgesic effects decide dose recommendations. Duloxetine can be used in the range 30–90 mg/day, while venlafaxine with less pronounced NA reuptake inhibition at low dosages needs a dose of at least 225 mg/day. Milnacipran is marketed for fibromyalgia and widespread body pain in the USA, but not in Europe. It can be an alternative if there are side effects limiting the use of the other SNRIs.

### Aminoketones

Bupropione lacks formal evidence for treatment of DGBIs, but its NA reuptake inhibition properties can be extrapolated as useful in parallel with the SNRIs. One alternative for use is as an augmentation (see below) in situations where fatigue and sleepiness are a dominant feature together with the gastrointestinal symptoms and a mood disorder [40].

### Selective Serotonin Reuptake Inhibitors

The selective serotonin reuptake inhibitors (SSRIs) do not have a NA effect and are thus generally not recommended when treatment of abdominal pain is the only indication. However, they are helpful for treating comorbid anxiety, depression, and psychological distress when clinically evident. In such instances, SSRIs may lead to improvement particularly for global symptom scores and should be used within traditional dose ranges for respective compound included in this class of drugs [2••]. Due to their side effect profile, SSRI treatment is recommended in situations where diarrhea is not a prominent feature. Starting at half the intended dose also reduces the risk for increased anxiety at initiation of SSRI treatment, sometimes also supplemented by a period of bensodiazepine treatment to overcome the risk for stopping prematurely due to a flare of anxiety symptoms.

### Tetracyclic Antidepressants

Mirtazapine is the drug with the best evidence for use in the context of DGBIs, more precisely in situations where functional dyspepsia with prominent features of postprandial distress syndrome (PDS) dominates or for chronic nausea/vomiting syndrome also associated with weight loss. Treatment with 15 mg/day improved overall symptom scores and resulted in weight gain in an 8-week study of patients with functional dyspepsia [41]. If comorbid depression is part of the illness spectrum associated with the DGBI, the indication for treatment is strengthened. Like for the TCAs, mirtazapine can improve a sleep disturbance as well [42]. Mianserin is an older agent with related mode of action that can be considered in DGBIs.

### Atypical Antipsychotics

In some situation with treatment refractory DGBI-related symptoms, the atypical antipsychotics are treatment options to consider. Olanzapine and quetiapine are the most studied where pain was associated with fibromyalgia, migraine, or other types of chronic headaches [43]. Compared to the older generation antipsychotics, these neuromodulators have less risk of extrapyramidal side effects and have in common an anxiolytic effect and sleep inducing effect, but have as side effects increased sedation and weight gain.

Quetiapine has a complex mode of action involving multiple receptors (D_2_, 5-HT_2A_, H_1_ receptor antagonism, partial 5-HT_1A_ receptor antagonism, and affinity for α_1_ and α_2_ receptors). One of its metabolites also has an effect as a NA reuptake inhibitor, which is of benefit for visceral analgesia [44]. The best evidence for treatment of pain comes from studies of fibromyalgia where quetiapine has been superior to placebo [45] but inferior to amitriptyline [46]. Another study has shown benefit of adding quetiapine to an SNRI or TCA for refractory gastrointestinal pain [47]. Its D_2_ receptor antagonism explains positive effects in the treatment of severe nausea and can be beneficial also when a disturbed sleep pattern is prominent due to its sedative effects. It is recommended to stay in the low-dose range (25–200 mg/day) when treating DGBIs. Olanzapine, a neuromodulator with strong 5-HT_3_ receptor-inhibiting effects apart from D_2_ receptor antagonism, is also useful particularly if chronic nausea and vomiting are the most troublesome symptoms in a DGBI. It can be considered as the second option to mirtazapine with experience from anesthesiology and oncology as the reference [48]. The olanzapine dose for this indication most often is (2.5–) 5–10 mg/day. Both quetiapine and olanzapine are an option if fibromyalgia is a comorbid feature in DGBIs.

Another group of atypical agents (aripiprazole, brexpiprazole) also have putative value in reducing anxiety and augmenting the pain benefits of antidepressants and lack the sedating or weight gain features of the previous agents. However, these medications are more likely to produce akathisia and other movement disorders though less so with the newer agent brexpiprazole [49].

### Azapirones

The azapirones (buspirone or tandospirone) are non-benzodiazepine antianxiety agents that also have effects on gastric accommodation (5-HT_1A_ agonism). They have both central and peripheral neuromodulator capacity. Significant symptom reductions have been shown after 4 weeks of treatment in patients with postprandial distress syndrome when using buspirone in the same dose range as for the treatment of anxiety (30 mg/day) [50]. Tandospirone is available only in China and Japan. Positive effects on abdominal pain ratings (≥ 50% reduction compared with baseline) among IBS patients were reported in a study confounded by simultaneous use of pinaverium [51], but it was not better than placebo in a study involving patients with functional dyspepsia [52]. With these data at hand, the clinical recommendation for buspirone is to use it when early satiety, fullness, and nausea dominate a DGBI.

## Augmentation Treatment

Finally, the concept of augmentation treatment, i.e., combining treatments in a clinical situation where individual therapeutic effects from the most common drugs presented above are either insufficient or complicated by side effects that restrict dosage to a suboptimal level, should be considered. Instead of totally abandoning one suboptimal neuromodulator, adding other neuromodulators, sometimes in a lower dosage to minimize the risk for side effects, can be useful. Knowledge about receptor affinities and peripheral versus central mode of actions together with dominant symptoms as combined selection grounds can result in additive effects. The formal evidence for augmentation treatment in DGBIs is lacking, but suggested from empirical grounds [53] and with experience from treatment of depression as the model [54••]. Examples of augmentation include adding an atypical antipsychotic or an azapirone to a TCA or SNRI, combining neuromodulator treatment with a behavioral intervention (e.g., hypnosis, CBT), adding a delta ligand agent to a TCA or SNRI particularly if there is a somatic component of pain or in some cases combining low-dose TCA with an SSRI.

**Fig. 2:**
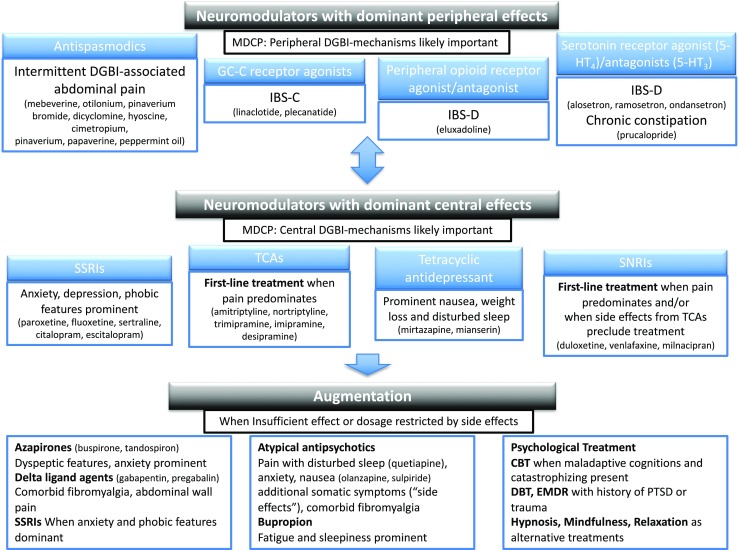
Conceptual summary of the different indications and clinical considerations in the selection of neuromodulating therapy within the framework of a multidimensional clinical profile (MDCP). Peripheral and central neuromodulators can be used on their own or in combinations depending on if central and peripheral mechanisms are judged as more or less important to the individual patient. When there are insufficient effects, or dosage is restricted by side effects, augmentation therapy can be applied with suggested combinations based on clinical features as given in the lower part of the figure. Note that non-pharmacologic treatment options should be considered (not covered in this article) as augmentation where this could be given also as an adjunct to peripheral neuromodulators given on their own when certain clinical features are present. DGBI disorders of gut-brain interaction, GCC guanylate cyclase-c, IBS-C irritable bowel syndrome with constipation, IBS-D irritable bowel syndrome with diarrhea, 5-HT 5-hydroxytryptamine (serotonin), SSRIs selective serotonin reuptake inhibitors, TCAs tricyclic antidepressants, SNRIs serotonin noradrenalin reuptake inhibitors, CBT cognitive behavioral therapy, DBT dialectic behavioral therapy, EMDR eye movement desensitization and reprocessing, PTSD post-traumatic stress disorder

A conceptual summary of indications and clinical recommendation when use of gut-brain modulators with peripheral and central actions is considered is given in Fig. 2, combinations suitable for augmentation included.

## Relapse Prevention

When it comes to termination of treatment with central modulators in patients that have had intense and long-standing symptomatology, there is not any good evidence for recommendation in DGBIs. For now, it is probably best advice to follow guidelines from major depressive disorders, i.e., continue treatment for at least 6–12 months after reaching a point of good response as rated by the patient, well aware of an increased risk of symptom relapse in the period that follows treatment termination [2••, 55, 56].

## Conclusions

The FGIDs, now called DGBIs, are often considered as difficult to treat and with use of pharmacologic treatment options of limited efficacy. However, newer research is showing the value of these treatments though the existing evidence is still scanty. Nevertheless, empiric evidence shows the value of neuromodulators in ameliorating symptom severity and improving quality of life. Included herein are a number of treatments that have reasonable value for accomplishing these management effects and also addressing sometimes multiple problems involved in the complex illness experience seen with DGBIs. The central role of gut-brain interactions is increasingly highlighted as most important to conceptually understand, also when trying to personalize treatments in these patients. The growing knowledge of neurogastroenterology and the use of neuromodulators are more than adequate to reduce the tendency for clinicians to say to patients, “I can not do any more, you just need to learn to live with it.” When intermittent symptoms of mild intensity are present, and with gut-related association (e.g., worse with eating, relieved by bowel movements) neuromodulators with peripheral actions most often are sufficient. But as the chronicity and intensity of symptoms become severe and dominant, particularly involving abdominal pain, nausea, or vomiting, along with extra-intestinal symptoms, one should consider starting or adding central neuromodulators to the existing treatments.
